# Epidemiology of drug-resistant tuberculosis in Chongqing, China: A retrospective observational study from 2010 to 2017

**DOI:** 10.1371/journal.pone.0216018

**Published:** 2019-12-10

**Authors:** Bo Wu, Ya Yu, Changting Du, Ying Liu, Daiyu Hu

**Affiliations:** 1 Outpatient Department, The Institute of Tuberculosis Prevention and Control, Chongqing, People’s Republic of China; 2 Department of Prevention and Control, The Institute of Tuberculosis Prevention and Control, Chongqing, People’s Republic of China; 3 Medical Administration Department, The Institute of Tuberculosis Prevention and Control, Chongqing, People’s Republic of China; Chinese Academy of Medical Sciences and Peking Union Medical College, CHINA

## Abstract

China is one of the top 30 countries with high multidrug-resistant tuberculosis (MDR-TB) and rifampin-resistant tuberculosis (RR-TB) burden. Chongqing is a southwest city of China with a large rural population. A retrospective observational study has been performed based on routine tuberculosis (TB) surveillance data in Chongqing from 2010 to 2017. The MDR/RR-TB notification rate increased from 0.03 cases per 100,000 population in 2010 to 2.1 cases per 100,000 population in 2017. The extensively drug-resistant TB (XDR-TB) notification rate has increased to 0.09 cases per 100,000 population in 2017. There was a decreasing detection gap between the number of notified MDR/RR-TB cases and the estimate number of MDR/RR-TB cases in new TB cases. The treatment success rate of MDR/RR-TB was 59% (95% confidence interval [CI], 53%-65%) in this period. Despite the progress achieved, the prevalence of MDR/RR-TB was still high facing challenges including detection gaps in new TB cases, the regional disparity, and the high risk for MDR/RR-TB in elderly people and farmers. Sustained government financing and policy support should be guaranteed in the future.

## Introduction

Drug-resistant tuberculosis (DR-TB) threatens global TB prevention and control. MDR-TB, defined as TB resistant to at least rifampin (RFP) and isoniazid (INH), has longer treatment regimens and lower treatment success rate with supplies of less effective and more toxic second-line drugs [1]. RR-TB, defined as TB resistant to at least RFP, also requires a second-line regimen, and 83% of RR-TB cases have MDR-TB [2].

From 2006 to 2014, China had implemented a project targeting MDR-TB through a partnership with the Global Fund to Fight AIDS, Tuberculosis, and Malaria (Global Fund) [3]. In 2009, the Bill and Melinda Gates Foundation and the Chinese Ministry of Health (now called the National Health Commission of China) announced the initiation of a comprehensive MDR-TB development programme aiming to provide innovative MDR-TB diagnosis tool, improve affordability of the treatment and the treatment success rate. A health system reform has also been launched since 2009 in China [4]. However, the prevalence of MDR-TB remains high. China is one of the 30 high MDR/RR-TB burden countries, and has an estimated 73,000 incident cases of MDR/RR-TB in 2016, accounting for 12.2% of all MDR/RR-TB burden in the world [2].

Chongqing is a municipality directly under the central government in the southwest of China with about 30 million people. Approximately 23,000 TB patients were reported in Chongqing in 2017, and the TB notification rate was 77.1 cases per 100,000 population which was 27.5% higher than the rate of the whole country. In 2009 and 2013, Chongqing participated in the programme of the Bill and Melinda Gates Foundation and the Global Fund respectively. Significant improvement in MDR/RR-TB notification, diagnosis and treatment has been achieved since 2009. In 2017, 643 MDR/RR-TB cases were notified in Chongqing, and the MDR/RR-TB notification rate is 2.1 cases per 100,000 population. In 2017, 29 cases with XDR-TB, defined as MDR-TB plus additional resistant to a fluoroquinolone and a second-line injectable, were notified in Chongqing. However, gaps between MDR/RR-TB notifications and the estimate number of MDR/RR-TB cases in new TB cases remains big. Since 2010, there have been about 4000 MDR/RR-TB cases undetected in Chongqing according to detection gap analysis in our study.

This observational retrospective study tried to explore the trend of MDR/RR-TB and the changes of drug resistance patterns based on routine surveillance data in Chongqing from 2010 to 2017. Our study may serve as a reference for the future policy of MDR/RR-TB control in Chongqing and other regions with similar situations.

## Methods

### Study design and data collection

This was an observational retrospective study of notified MDR/RR-TB cases from 2010 to 2017 in Chongqing. The information of MDR/RR-TB cases came from the national electronic TB surveillance system.

The DR-TB notification rate from 2010 to 2017 was analyzed, including MDR/RR-TB and XDR TB. Through this analysis, we could see whether the improvement of detection and diagnosis in recent years had promoted the notification of MDR/RR-TB in Chongqing. The MDR/RR-TB cases were stratified by age, sex, and occupation for trend analysis. Though this analysis, we could know the local populations that should be focused on in MDR/RR-TB prevention and control. The information of sex, age and occupation of MDR/RR-TB cases came from the national TB surveillance system. The socio-demographic data came from Chongqing Statistical Yearbook from 2010 to 2017. According to the Chongqing Statistical Yearbook, the population in Chongqing was divided into four age groups: 0–17 years, 18–34 years, 35–59 years, and over-60 years. The occupation of MDR/RR-TB cases was divided into farmers and non-farmers. The regional disparity of TB notification has also been evaluated. According to the division of local government, there were four regions in Chongqing including: the Urban Districts, New Urban Development Districts, Northeast Districts and Southeast Districts. These regions were different in terms of socioeconomic development. The geographic information of different regions was analysed using the map feature of Epi Info (Epi Info Version 7.2, The Centers for Disease Control and Prevention, Atlanta, GA, USA).

The detection gap between the number of notified MDR/RR-TB cases and the estimated number of MDR/RR-TB cases has been evaluated. In ideal status, every bacteriological positive TB case should have undergone sputum culture, and the number of TB cases who should have undergone sputum culture should equal the number of notified bacteriological positive TB cases in the process of estimating the number of MDR/RR-TB cases. Bacteriological positive TB cases were notified from sputum smear test or rapid molecular test. According to the national DR-TB survey in 2013, the MDR/RR-TB proportion in new TB cases was 7.1% and the MDR/RR-TB proportion in previously treated TB cases was 24% [2]. The two percent could be used to multiply the number of notified bacteriological positive TB cases who should have undergone sputum culture in new TB cases and in previously treated TB cases respectively, and the estimated number of MDR/RR-TB cases could be calculated. A new TB case was defined as a patient who has never been treated or been treated less than 1 month, a treated case was defined as a patient who previously received TB treatment for 1 month or more previously [5,6].

The information of MDR/RR-TB screening, Drug susceptible test (DST), diagnosis, treatment, management and outcome has been recorded in the national electronic surveillance system since 2010. The data of MDR/RR-TB cases from 2010 to 2017 came from this system. Reporting of extra-pulmonary TB was not mandatory in this system, thus all data were based on the pulmonary MDR/RR-TB cases. Population and socioeconomic data came from Chongqing Statistical Yearbook from 2010 to 2017.

### Risk factor analysis

To explore risk factor associated with MDR/RR-TB in Chongqing, we have analyzed potential risk factors including age, sex, occupation, region, and treatment history. MDR/RR-TB screening data from 2010 to 2017 were analyzed, and MDR/RR-TB cases have been compared with drug-susceptible culture-confirmed TB cases.

### Drug resistance patterns

DST include both phenotypic (conventional) and genotypic (molecular) testing methods. In Chongqing, conventional DST was performed at the provincial reference laboratory using the proportion method on acid-buffer Lowenstein-Jensen (L-J) Medium. The first-line drugs tested for drug-resistance included INH, RFP, ethambutol (EMB), and streptomycin (SM). There were two second-line drugs reported, including ofloxacin (OFX) and kanamycin (KM). Rapid molecular test was performed at capable county-level laboratories and prefectural laboratories. In Chongqing, the main rapid molecular test included Xpert MTB/RIF and fluorescence PCR melting curve method. Both rapid molecular tests could detect INH and RFP resistance in one day. External quality assessment has been conducted through national reference laboratory of China. In the analysis of drug resistance patterns, we enrolled only the culture-positive cases whose clinical isolates were performed with phenotypic DST for all the six drugs.

### The treatment outcome

According to definitions and reporting framework for tuberculosis of World Health Organization (WHO) [5], the treatment outcome from 2010 cohort to 2015 cohort was also analyzed. Treatment success was the sum of cured and treatment completed. In the process of calculating the treatment success rate, we have enrolled only the MDR/RR-TB cases who were involved in treatment, and we have excluded the MDR/RR-TB cases who had not treatment outcome information reported. We also excluded the MDR/RR-TB cases loss to follow-up. We have implemented missing data analysis and multiple imputation analysis in SPSS.

### Detection and treatment procedure

In Chongqing, the MDR/RR-TB control system was constructed of three levels: province, prefecture, and county. Provincial TB prevention and control institution was responsible for administration, supervision, DST, and surveillance.

There were 38 counties and districts in Chongqing, every one of which had a county-level designated clinic for TB. The county-level designated clinics detected TB cases according to the tuberculosis diagnostic criteria WS288-2008 of China [7], and screened MDR/RR-TB.

In early stages, inadequate resources limited the systematic development of screening. Screening had been conducted spontaneously in all counties and districts before 2010. With the introduction of some projects, screening was becoming more and more standardized. Since 2013, screening in all counties and districts has supported by local government investment and the Global Fund. MDR/RR-TB suspects stemmed from notified bacteriological positive TB cases.

Since 2010, more and more county-level laboratories had been furnished with rapid molecular test equipment. In the counties and districts furnished with rapid molecular test equipment, rapid molecular test was conducted firstly. When the result of rapid molecular test was positive, sputum culture was conducted too. In the counties and districts without rapid molecular test equipment, sputum smear and culture was conducted. If a positive culture result was confirmed, the strain of MDR/RR-TB suspect would be sent to the provincial reference laboratory for strain identification and DST for six drugs.

After diagnosis by a prefectural expert review committee, most MDR/RR-TB cases were enrolled into a standardized regimen. MDR/RR-TB cases stayed 2 months in the prefectural designated hospital, and then received an ambulatory treatment course for 22 months managed by the county-level center for disease control and prevention (CDC) or county-level TB prevention and control institution.

### Ethics approval

The study was approved by the Ethics Committee of the Institute of Tuberculosis Prevention and Control, Chongqing, China. A secondary analysis based on non-identifiable data has been conducted and informed consent from individuals was not required. All methods were performed in accordance with relevant guidelines and regulations.

### Statistical analysis

The chi-square test for linear trend was used to test the trend of the notification rate based on annual data between 2010 and 2017. A Poisson model was performed, which was based on the counts of cases with the population as a denominator and regression equation for characteristics such as sex, age, occupation and region. A logistic regression model was performed to assess association between notification and risk factors. The missing data analysis for treatment outcome was implemented. A two-sided P-value less than 0.05 was taken as statistically significant. Statistical analyses were performed with the SPSS 22.0 software (SPSS, Inc., Chicago, IL, USA).

## Results

### Trend of MDR/RR-TB notification

**Fig 1:**
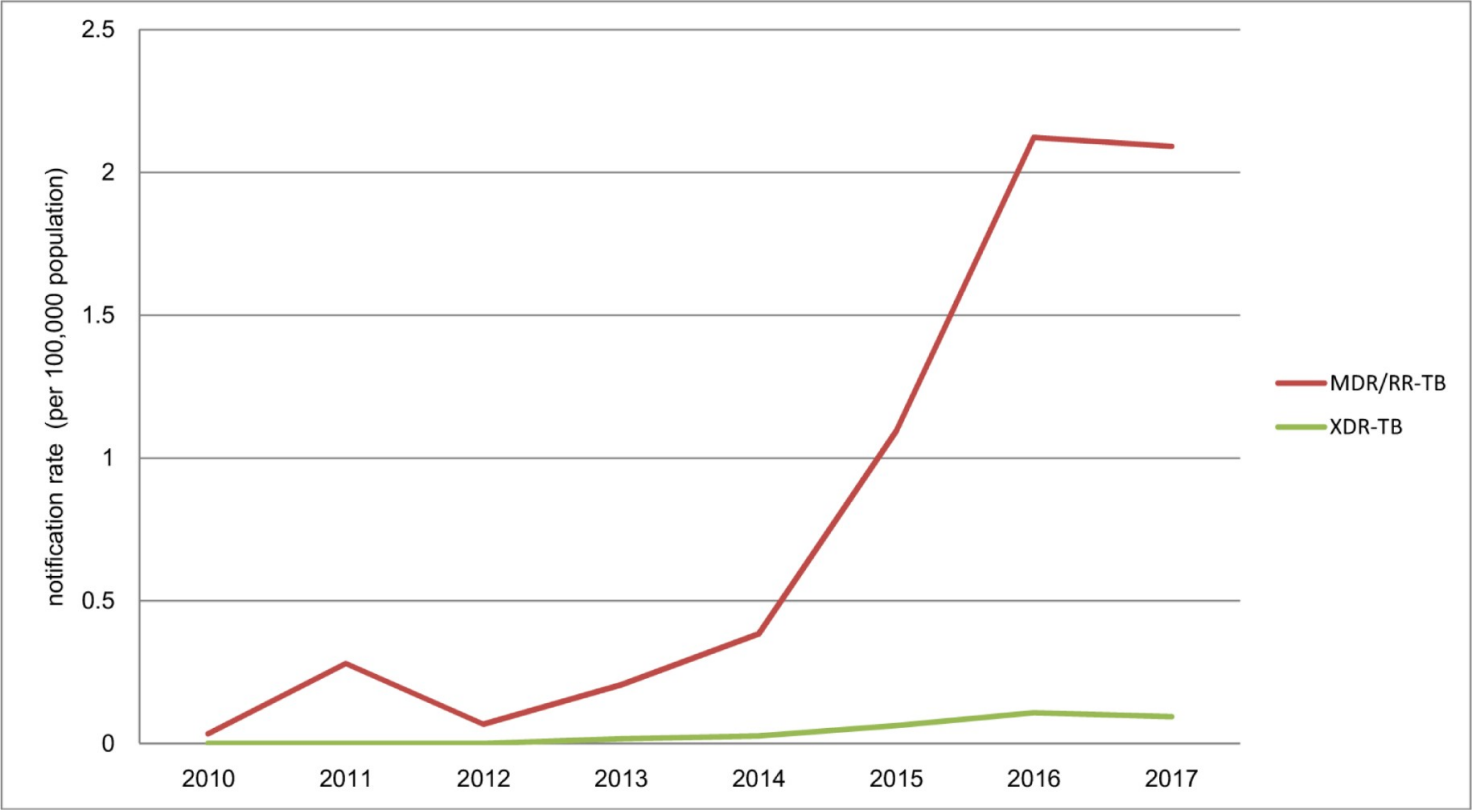
The trend of DR-TB notification rate from 2010 to 2017.

From 2010 to 2017, 1,908 MDR/RR-TB cases were notified in Chongqing. Among them, 4.9% were XDR-TB cases. The MDR/RR-TB notification rate increased significantly from 0.03 cases per 100,000 population in 2010 to 2.1 cases per 100,000 population in 2017 (χ^2^ trend = 1,623.2, *P*<0.001) (Fig 1). The XDR-TB notification rate increased significantly from 0.02 cases per 100,000 population in 2003 to 0.09 cases per 100,000 population in 2017 (χ^2^ trend = 27.1, *P*<0.001).

### MDR/RR-TB notification stratified by sex, age, and occupation

From 2010 to 2017, 70% of notified MDR/RR-TB cases were male. The MDR/RR-TB notification rate of male was significantly higher than the rate of female (OR, 2.2; 95% CI, 2–2.4). MDR/RR-TB notification rate of male increased significantly from 0.04 cases per 100,000 population in 2010 to 2.6 cases per 100,000 population in 2017 (χ^2^ trend = 1,133.7, *P*<0.001). MDR/RR-TB notification rate of female also increased significantly from 0.02 cases per 100,000 population in 2010 to 1.1 cases per 100,000 population in 2017 (χ^2^ trend = 536.8, *P*<0.001).

**Fig 2:**
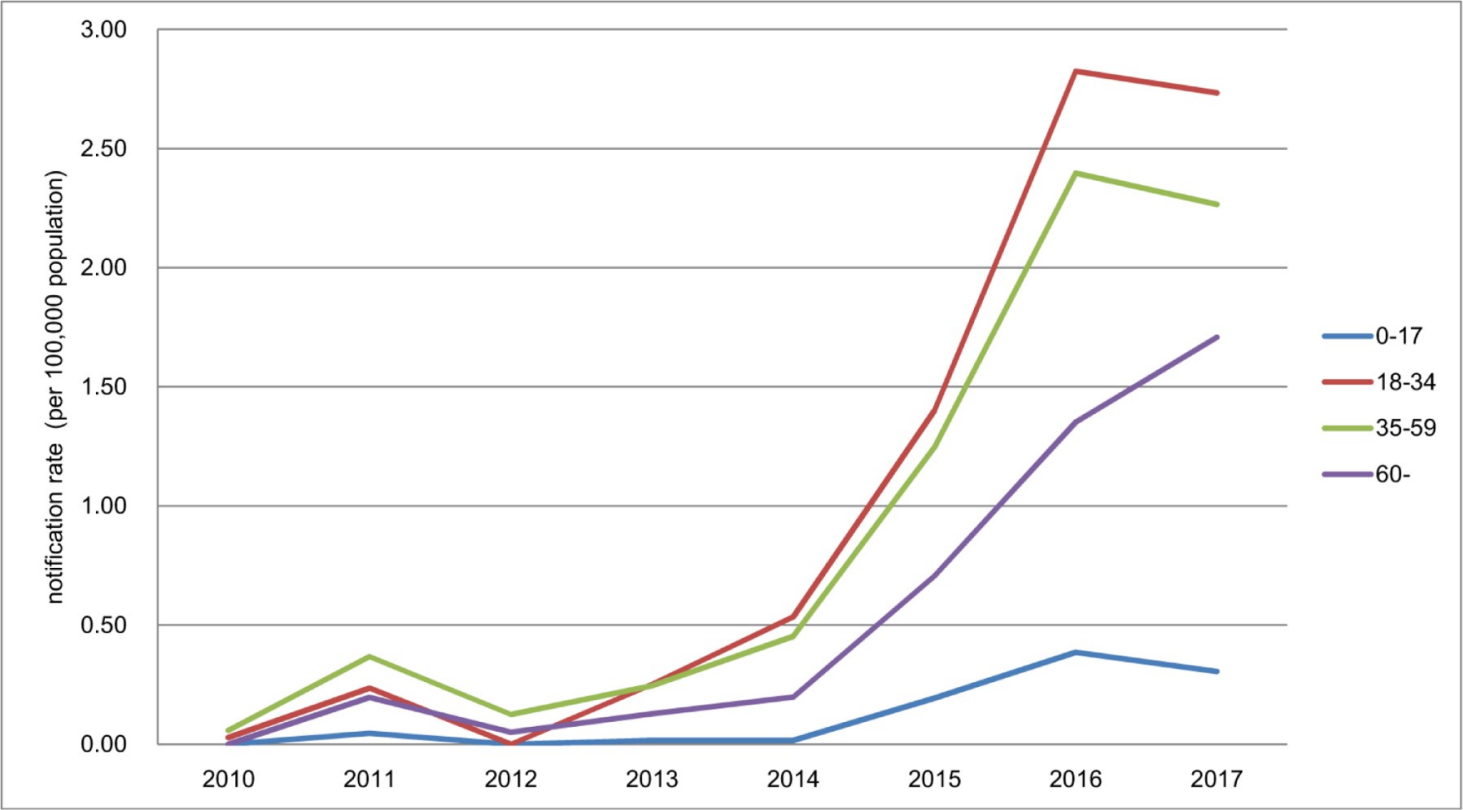
The MDR/RR-TB notification rate in different age groups from 2010 to 2017.

From 2010 to 2017, the trend of age-specific MDR/RR-TB notification rate increased significantly among all age groups. The MDR/RR-TB notification rate of over-60-year age group (OR, 1.5; 95% CI, 1.2–1.8) was significantly lower than the rate of 18–34 age group (OR, 2.1; 95% CI, 1.9–2.4). The MDR/RR-TB cases aged 18–34 and 35–59 accounted for 81% of all notified MDR/RR-TB cases. From 2010 to 2017, the MDR/RR-TB notification rate in the age group 18–34 years had a significant increase from 0.03 to 2.7 cases per 100,000 population (χ^2^ trend = 585.3, *P*<0.001). The MDR/RR-TB notification rate in the age group 35–59 years also increased from 0.06 to 2.3 cases per 100,000 population (χ^2^ trend = 764.9, *P*<0.001) in this period. The MDR/RR-TB cases aged 0–17 had the lowest notification rate, and the notification rate increased significantly from 0 to 0.3 cases per 100,000 population (χ^2^ trend = 61.9, *P*<0.001). The proportion of the notified MDR/RR-TB cases aged 0–17 was also lowest, which was 3.1%. The MDR/RR-TB notification rate of over-60-year age group increased significantly from 0 to 1.7 cases per 100,000 population (χ^2^ trend = 264.7, *P*<0.001) (Fig 2).

The MDR/RR-TB notification rate of farmers was significantly higher than the rate of all other occupations from 2010 to 2017 (OR, 3.2; 95% CI, 2.9–3.5). From 2010 to 2017, the MDR/RR-TB notification rate of farmers has significantly increased (χ^2^ trend = 499.8, *P*<0.001).

### Regional disparity of MDR/RR-TB notification

**Fig 3:**
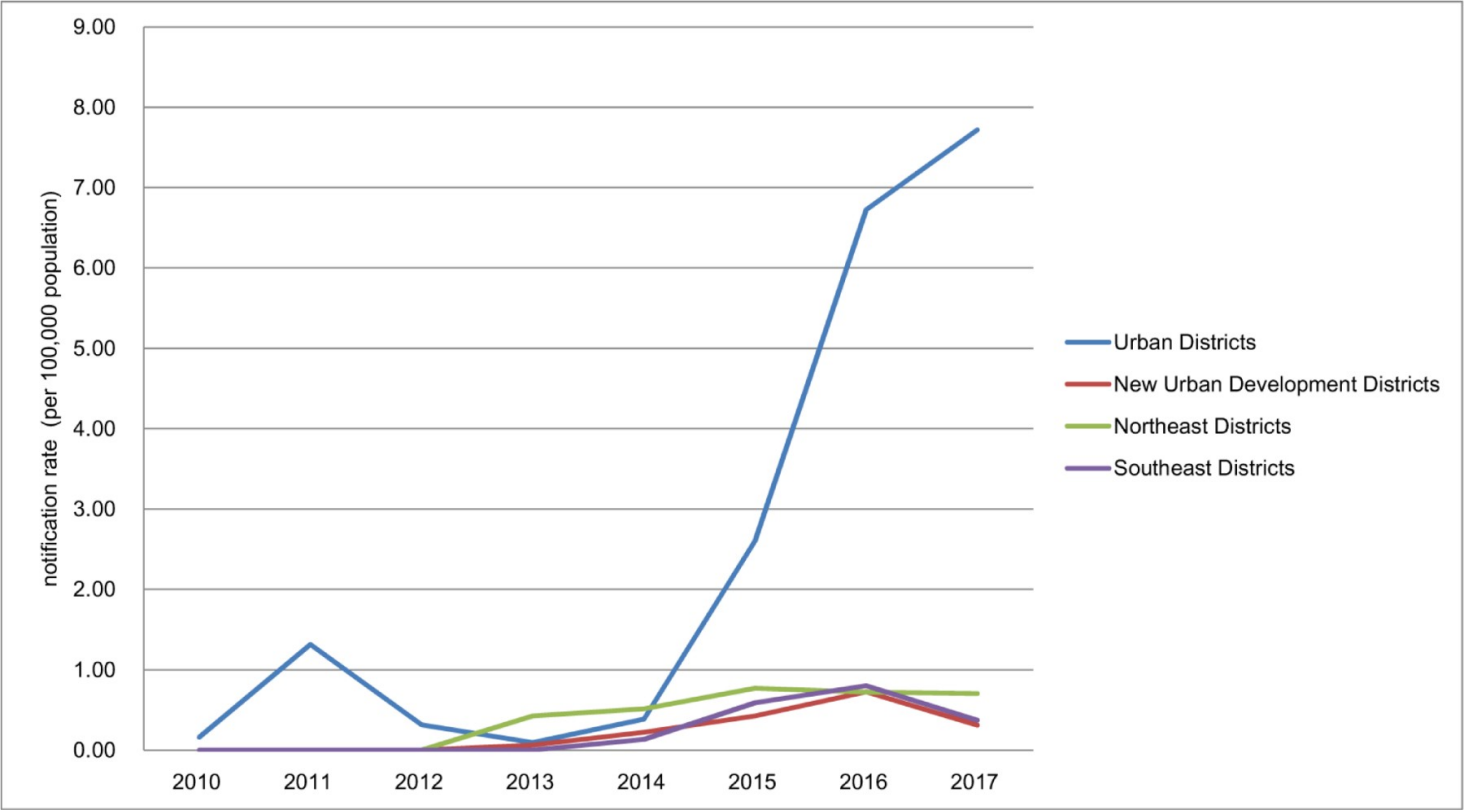
The MDR/RR-TB notification rate of different regions from 2010 to 2017.

**Fig 4:**
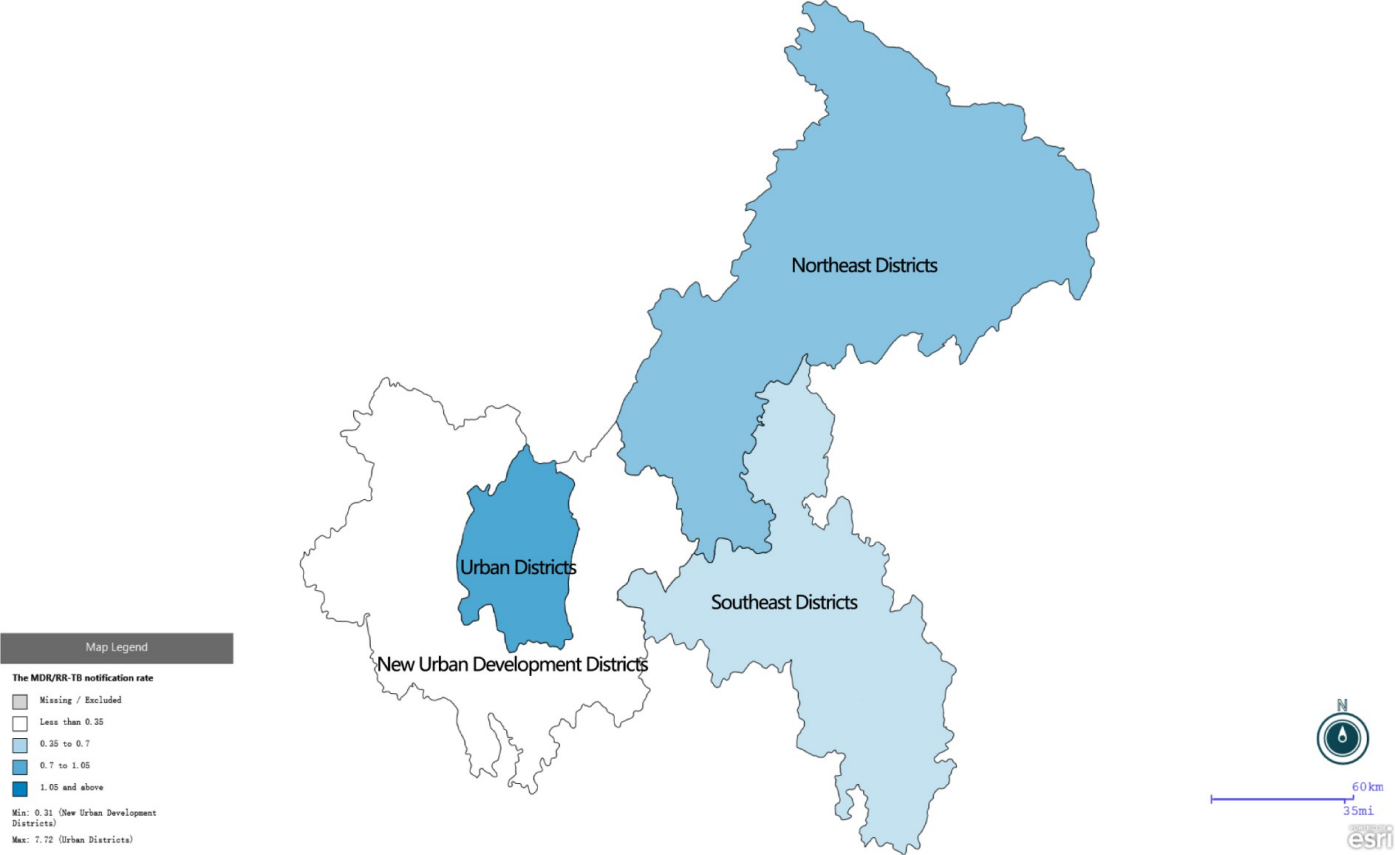
The MDR/RR-TB notification rate of different regions in 2017.

The MDR/RR-TB notification rate of Urban Districts (OR, 10.2; 95% CI, 8–12.9) and Northeast Districts (OR, 1.7; 95% CI, 1.3–2.1) was significantly higher than the rate of Southeast Districts. The MDR/RR-TB notification rate of New Urban Development Districts was not significantly different with the rate of Southeast Districts (OR, 0.92; 95% CI, 0.71–1.2) (Fig 3). The MDR/RR-TB notification rate of Urban Districts has significantly increased from 0.16 to 7.7 cases per 100,000 population (χ^2^ trend = 1,196.8, *P*<0.001). In other three regions, The MDR/RR-TB notification rate was less than 1 case per 100,000 population. The MDR/RR-TB notification rate in different regions in 2017 was shown in a map (Fig 4).

### Detection gap

**Fig 5:**
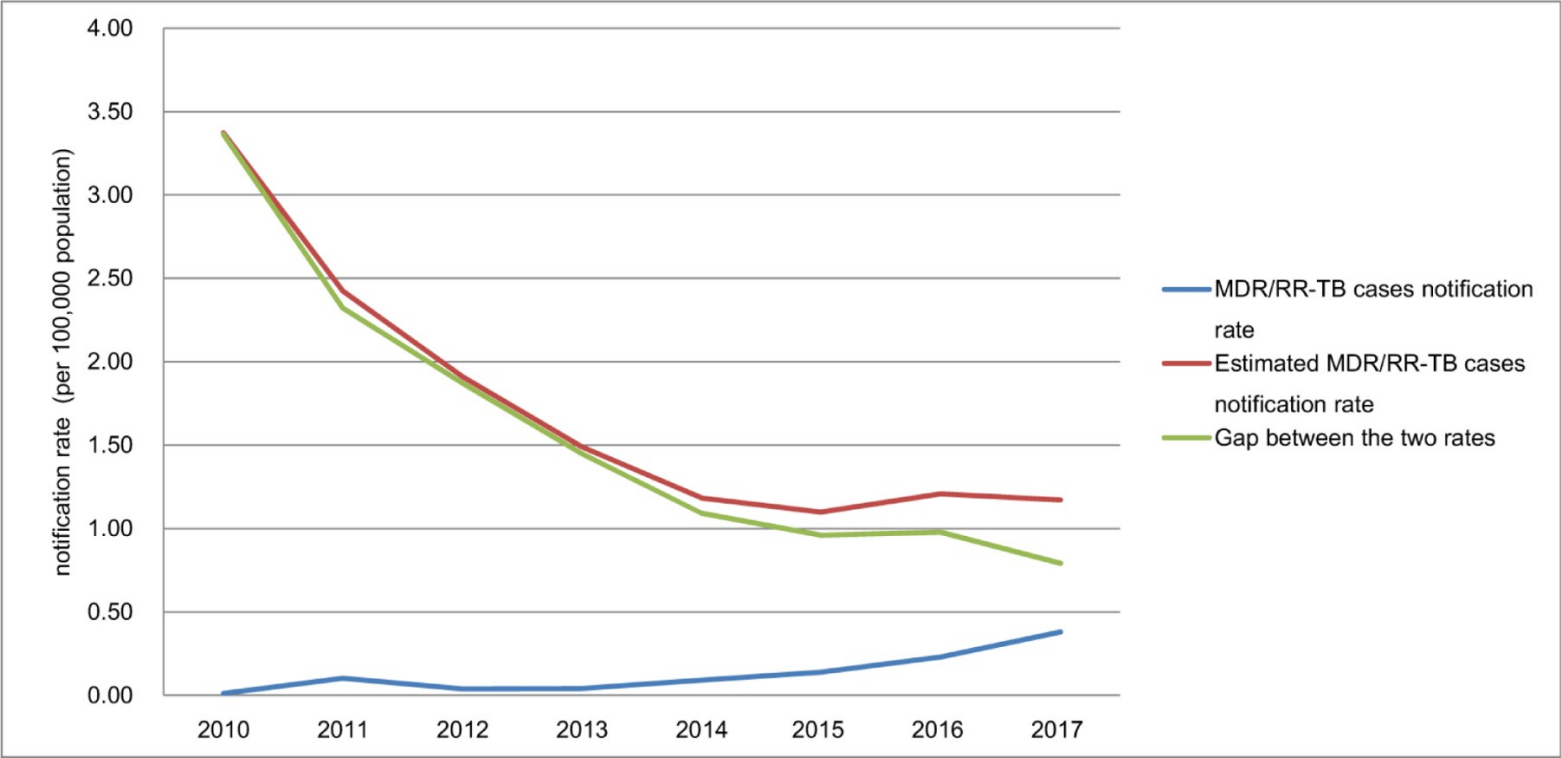
The notification gaps in new TB cases from 2010 to 2017.

The estimated number of MDR/RR-TB cases among TB cases who should have undergone sputum culture from 2010 to 2017 was 5901, which was 309% higher than the number of notified MDR/RR-TB cases. The detection gap between the number of notified MDR/RR-TB cases and the estimated number of MDR/RR-TB cases among TB cases who should have undergone sputum culture has significantly declined from 3.4 to 0.79 cases per 100,000 population in new TB cases from 2010 to 2017 (χ^2^ trend = 1,006.5, *P*<0.001) (Fig 5).

**Fig 6:**
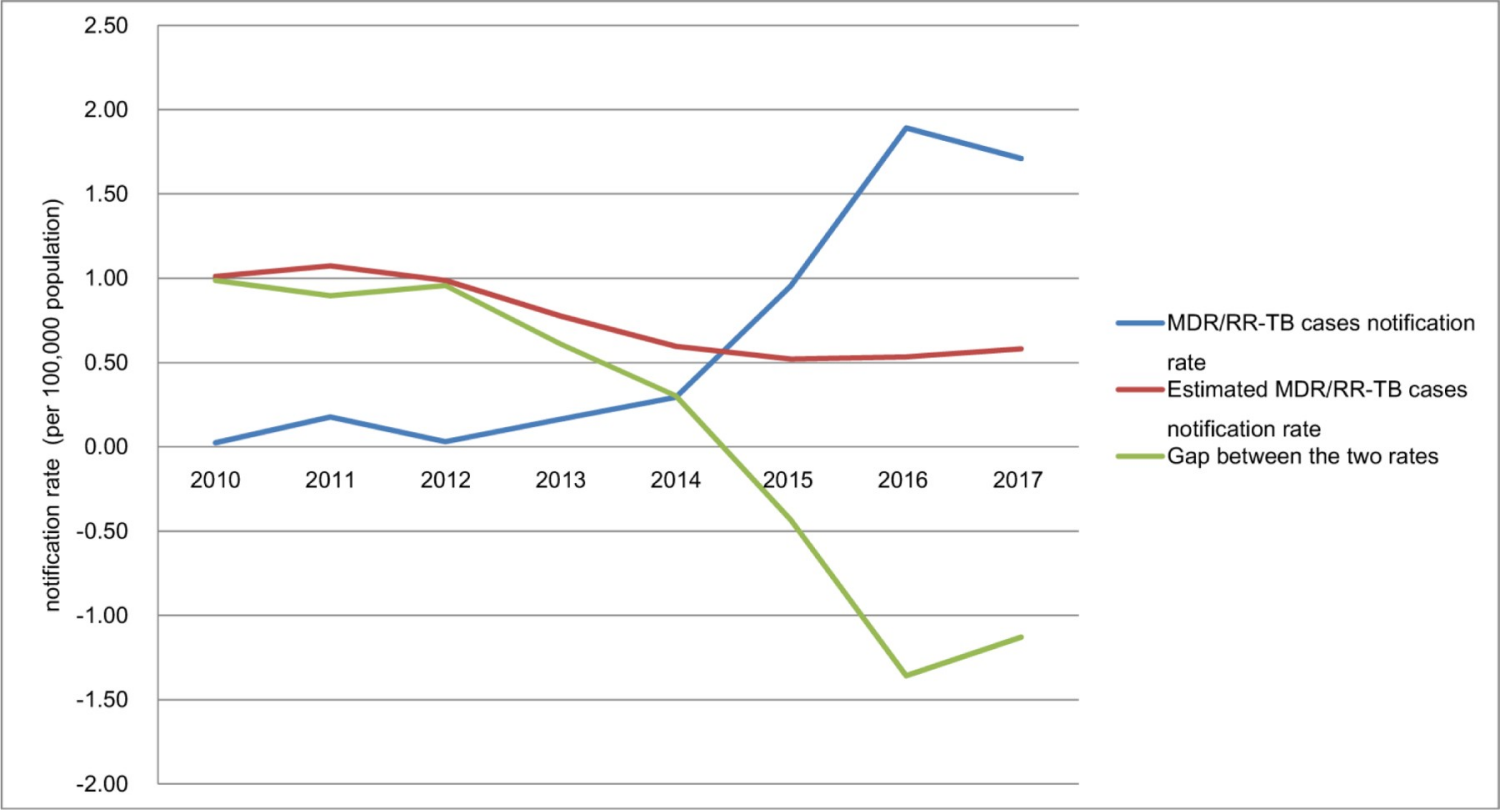
The notification gaps in previously treated TB cases from 2010 to 2017.

The detection gap has significantly declined from 1 to 0.3 cases per 100,000 population in previously treated TB cases from 2010 to 2014 (χ^2^ trend = 135.6 *P*<0.001), and the MDR/RR-TB cases notification rate has surpassed the estimated MDR/RR-TB cases notification rate since 2015 (Fig 6).

### Treatment outcome

From 2010 to 2015, there were 618 MDR/RR-TB cases diagnosed in Chongqing. Among them, 132 MDR/RR-TB cases were not involved in the treatment mainly because of poverty. There were 486 MDR/RR-TB cases who began the treatment. Among them, 42 were lost to follow up, and 124 had not treatment outcome information reported. So there were 132 MDR/RR-TB cases were missed before the treatment, and 166 MDR/RR-TB cases were missed in the treatment.

We have implemented missing data analysis in SPSS. In the diagnosed 618 MDR/RR-TB cases, the mean age was 41.2 (95% CI, 39.6–42.8) in the 320 MDR/RR-TB cases with treatment outcome information reported, and it was 43.2 (95% CI, 41.5–44.9) in the 298 MDR/RR-TB cases who were not involved in the treatment, lost to follow up or without treatment outcome information reported. The mean age was not significantly different in two groups (*Z* = 1.7, *P* = 0.09).

Among the 618 MDR/RR-TB cases, 50% of male MDR/RR-TB cases and 43% of female MDR/RR-TB cases were missed, and there were 50% of farmers and 46% of non-farmers who were missed.

**Fig 7:**
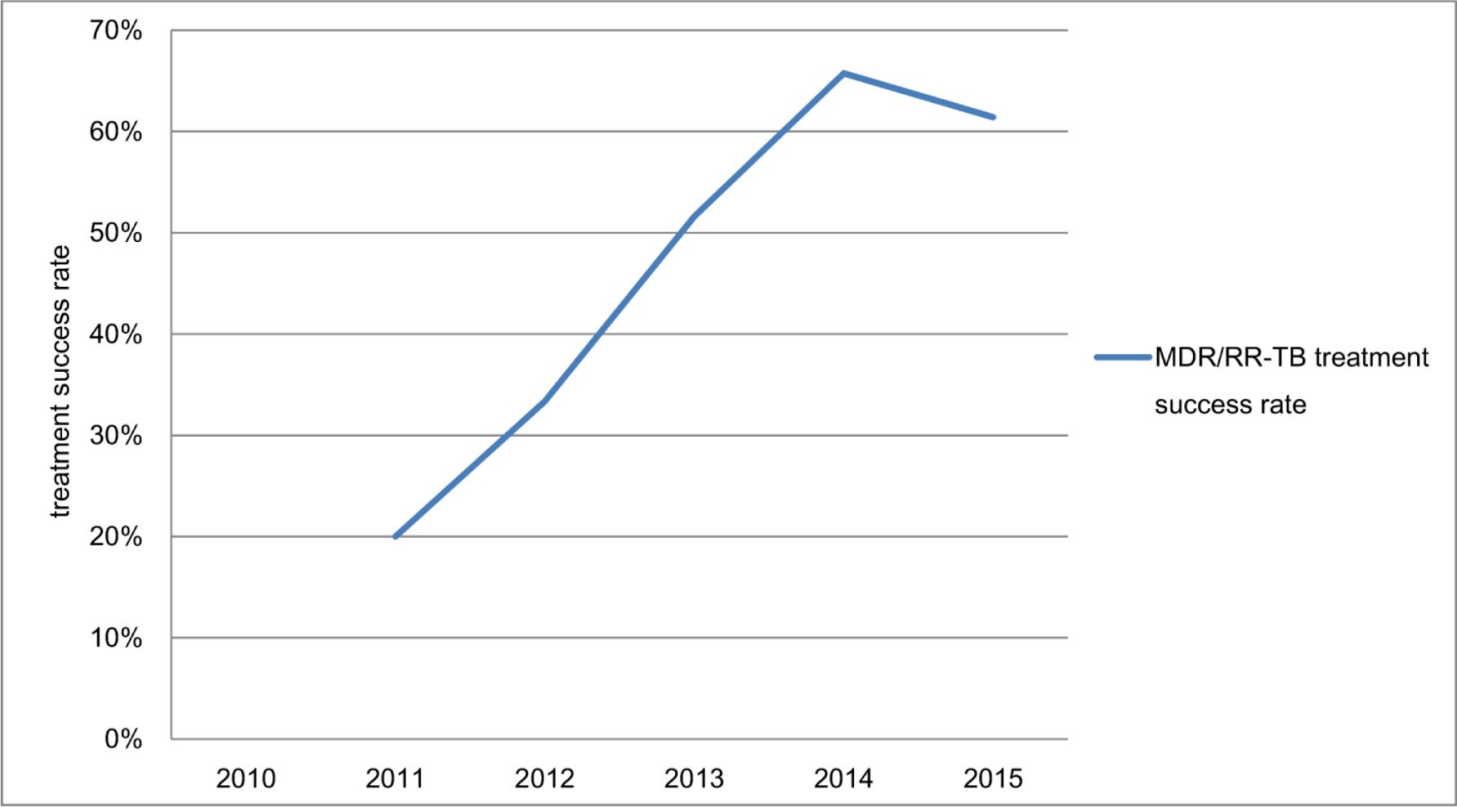
The MDR/RR-TB treatment outcome without imputed missing data.

According to the data before imputation, the treatment success rate of MDR/RR-TB was 59% (95% CI, 53%-65%) in this period. The cure rate, the death rate and treatment failure rate were 37.8% (95% CI, 31.9%-44%), 15.7% (95% CI, 11.2%-20.1%), and 10.7% (95% CI, 6.9%-14.5%) respectively (Fig 7).

We imputed the missing data in SPSS. According to the imputed data, the treatment success rate of MDR/RR-TB was 59% (95% CI, 57%-62%) in this period. The cure rate, the death rate and treatment failure rate were 37.5% (95% CI, 35.4%-39.7%), 16.2% (95% CI, 14.6%-17.8%), and 10.2% (95% CI, 8.9%-11.6%) in this period respectively.

**Fig 8:**
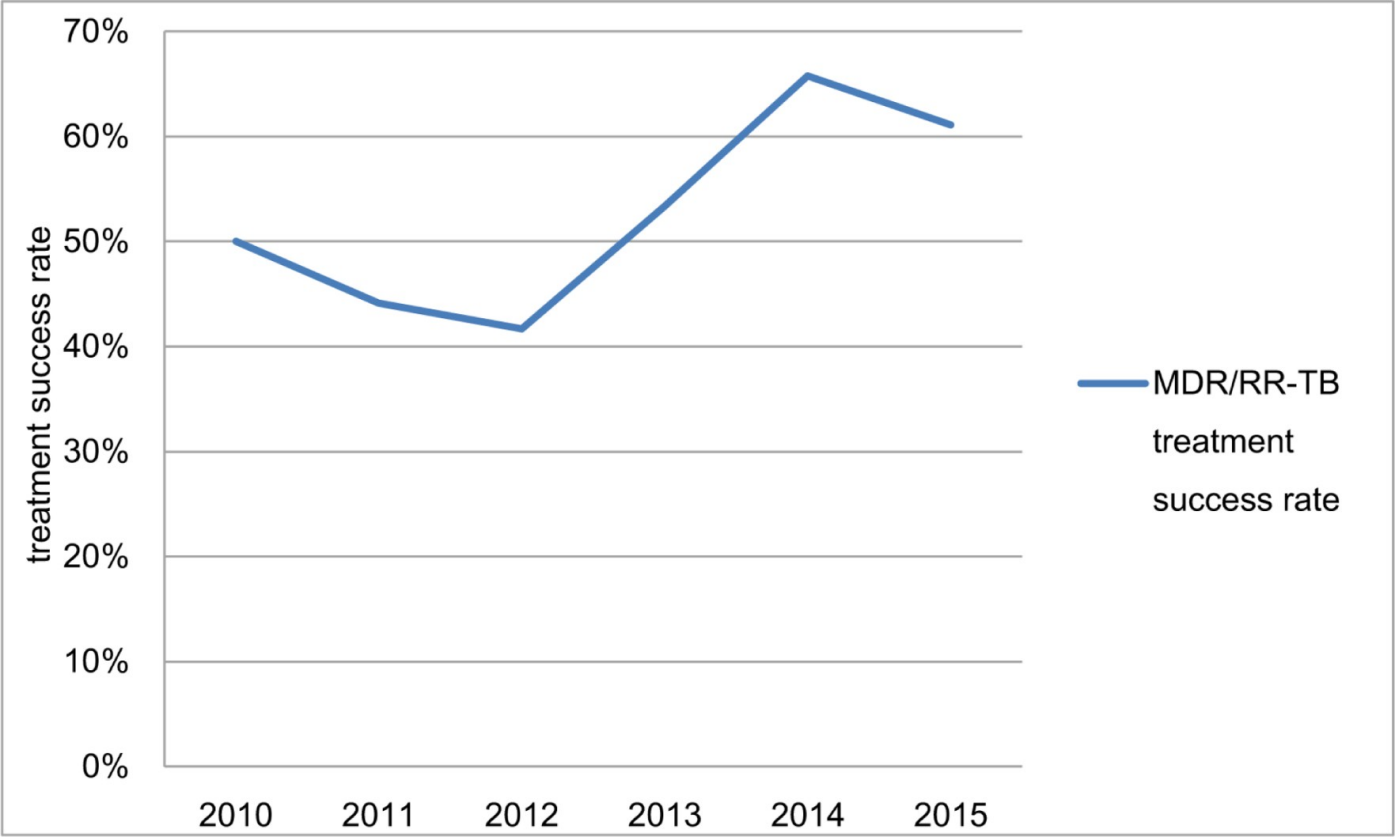
The MDR/RR-TB treatment outcome with imputed missing data.

The treatment success rate of MDR/RR-TB increased significantly from 50% (95% CI, 12.3%-88%) in 2011 cohort to 61% (95% CI, 58%-64%) in 2015 cohort (*χ2* trend = 23.8, *P*<0.001) (Fig 8).

### Drug resistance patterns

**Fig 9:**
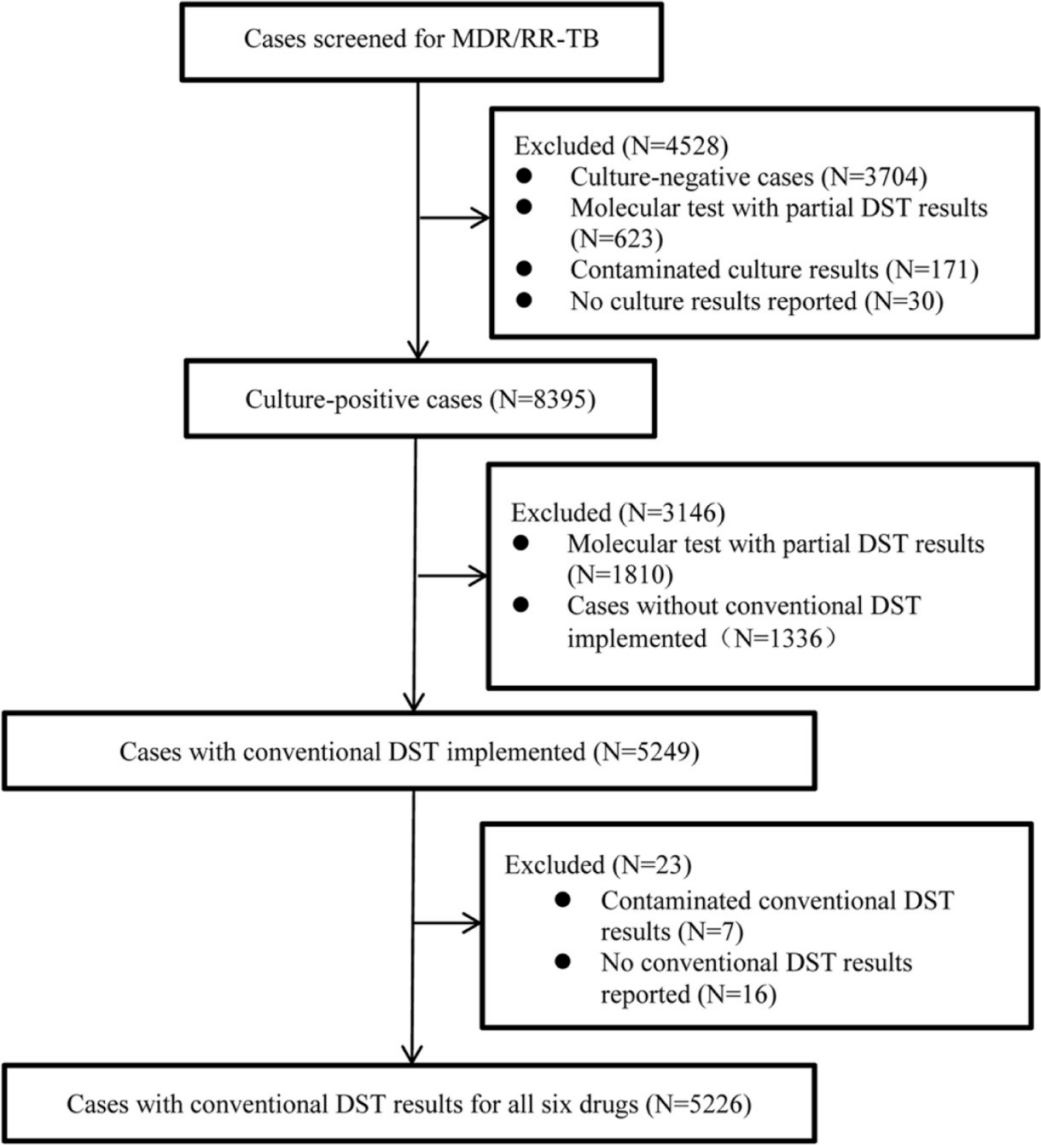
The flow chart of cases tested with conventional DST from 2010 to 2017.

From 2010 to 2017, there were 5226 cases tested with conventional DST in Chongqing (Fig 9). The rate of drug resistance for RFP in new TB cases was 9% (95% CI, 8.1%-10%), and this rate in previously treated TB cases was 41.5% (95% CI, 39%-44%).

INH had the highest rate of drug resistance which was 23.2% (95% CI, 22.1%-24.4%), followed by RFP (21.4% [95% CI, 20.3%-22.5%]), SM (14.6% [95% CI, 13.6%-15.5%]), EMB (9.9% [95% CI, 9.1%-10.7%]), OFX (9.5% [95% CI, 8.7%-10.3%]), and KM (2.4% [95% CI, 2.%-2.9%]). The drug resistance rate for RFP increased significantly from 7.1% (95% CI, 1.9%-12.2%) in 2010 to 25.9% (95% CI, 23.3%-28.5%) in 2017 (χ^2^ trend = 450.7, *P*<0.001). The drug resistance rate for INH had a similar trend which increased significantly from 13.1% (95% CI, 6.4%-20%) in 2010 to 26.6% (95% CI, 24%-29.2%) in 2017 (χ^2^ trend = 485.4, *P*<0.001).

**Fig 10:**
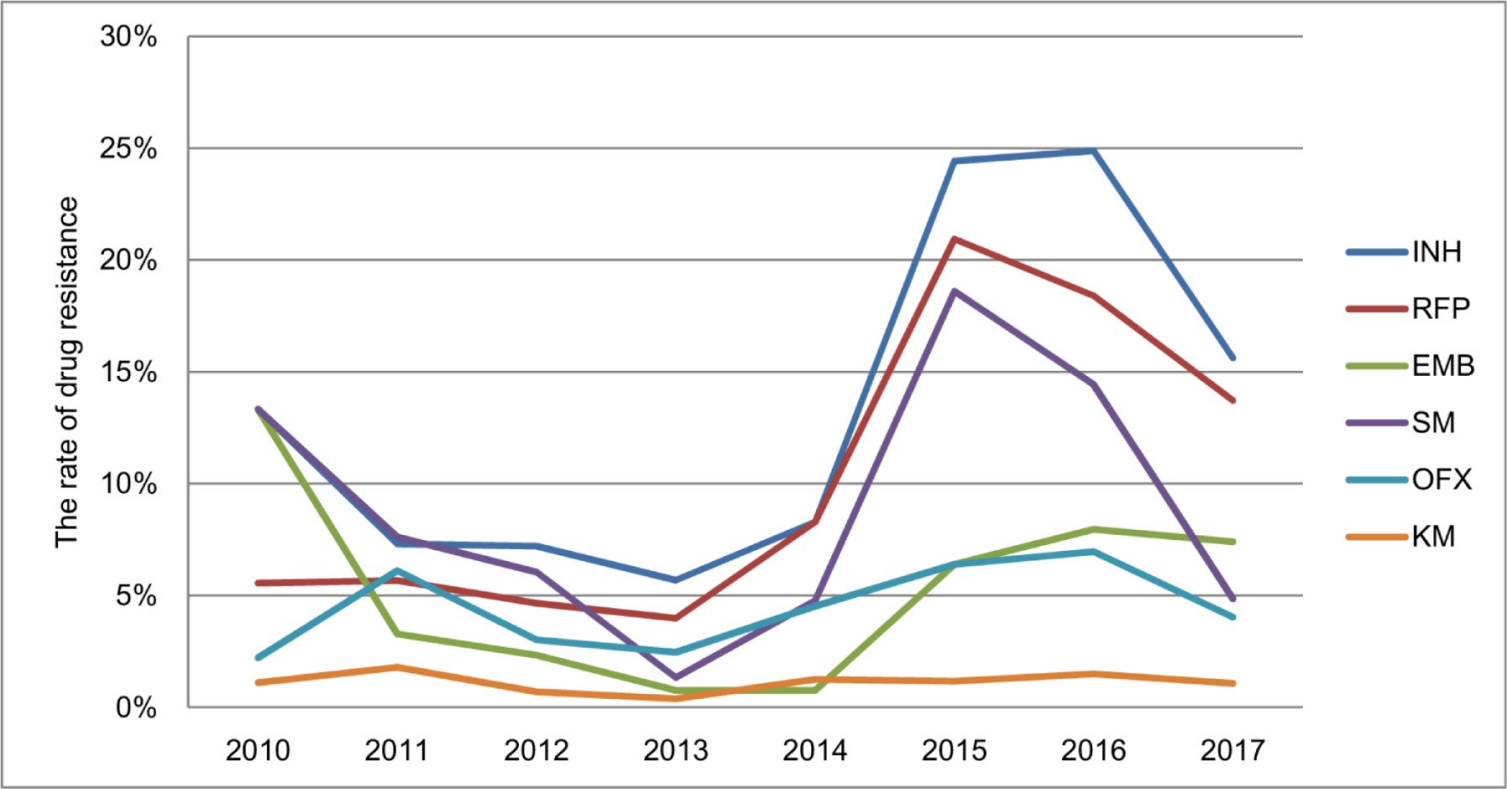
The drug resistance patterns in new TB cases from 2010 to 2017.

Among new TB cases screened, INH had the highest rate of drug resistance which was 11.2% (95% CI, 10.1%-12.3%) from 2010 to 2017, followed by RFP (9% [95% CI, 8%-10%]), SM (6.6% [95% CI, 5.7%-7.4%]), OFX (4.4% [95% CI, 3.7%-5.1%]), EMB (4.1% [95% CI, 3.4%-4.8%]), and KM (1.1% [95% CI, 0.75%-1.5%]). The drug resistance rate for RFP increased significantly from 5.6% (95% CI, 0.7%-10.4%) in 2010 to 13.7% (95% CI, 11.3%-16.2%) in 2017 (χ^2^ trend = 108.3, *P*<0.001). INH showed a significant rising trend (χ^2^ trend = 119.3, *P*<0.001) (Fig 10).

**Fig 11:**
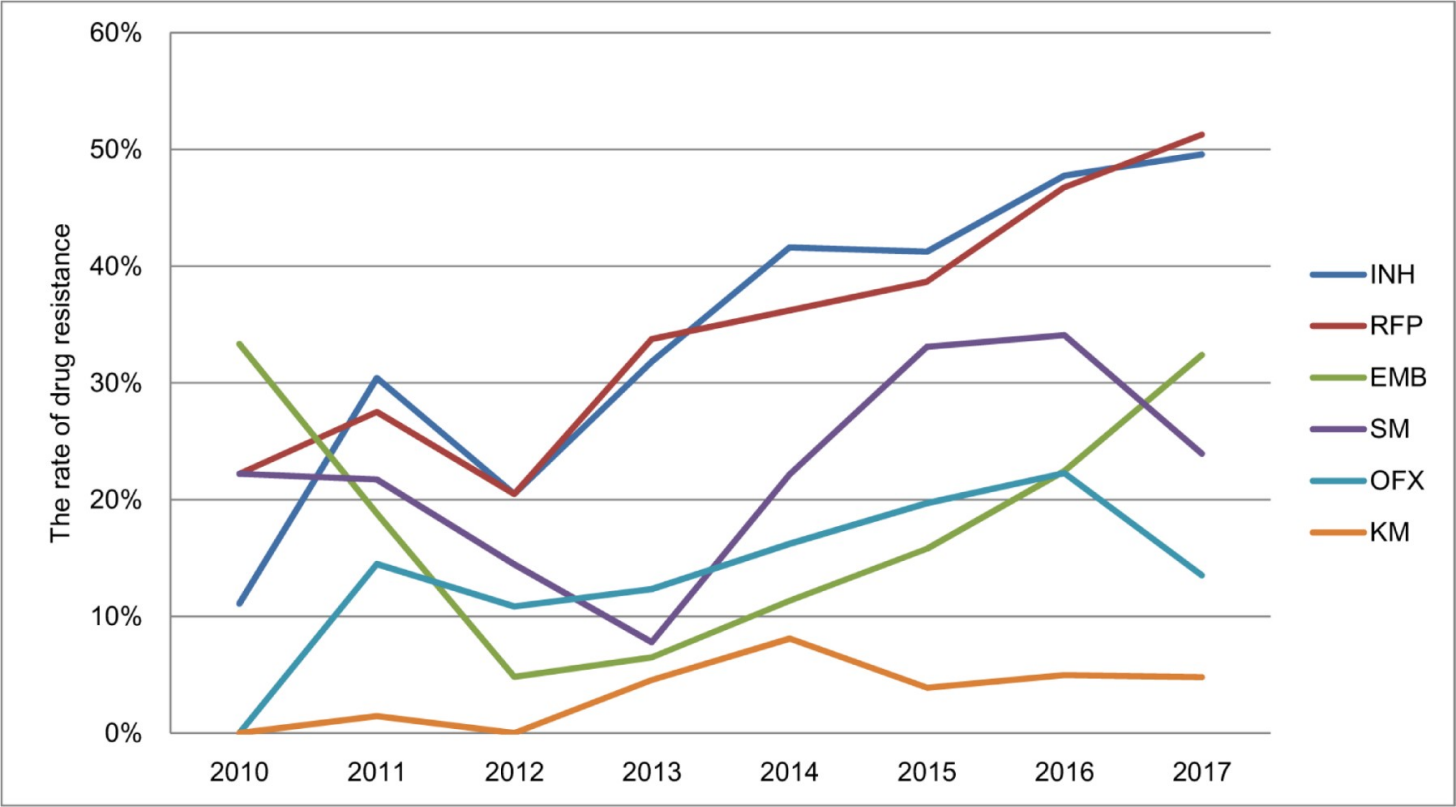
The drug resistance patterns in previously treated TB cases from 2010 to 2017.

Among previously treated TB cases screened, INH had the highest rate of drug resistance which was 43% (95% CI, 41%-45%) from 2010 to 2017, followed by RFP (41.5% [95% CI, 39.3%-43.7%]), SM (27.6% [95% CI, 25.6%-29.6%]), EMB (19.4% [95% CI, 17.6%-21.1%]), OFX (17.6% [95% CI, 16.2%-19.5%]), and KM (4.6% [95% CI, 3.7%-5.5%]). The drug resistance rate for RFP increased significantly from 22.2% (95% CI, 11.7%-56%) in 2010 to 51% (95% CI, 46%-56%) in 2017 (χ^2^ trend = 50.5, *P*<0.001). INH also showed a significant rising trend (χ^2^ trend = 45.8, *P*<0.001) (Fig 11).

### Risk factors associated with MDR/RR-TB

The risk factors associated with MDR/RR-TB were analyzed (Table 1). In single variable analysis, some groups of TB cases were more likely to develop MDR/RR-TB, including male, farmer, TB cases aged over-60 years, and previously treated TB cases. TB cases in Urban Districts were less likely to suffer MDR/RR-TB, but TB cases in New Urban Development Districts and Northeast Districts had a greater likelihood of MDR/RR-TB.

**Table 1:** Risk factors associated with MDR/RR-TB.

factors	Single variable analysis			Multiple variable analysis		
	cOR	95% CI	P	aOR	95%CI	P
**Gender (Compared with "Female")**						
**Male**	1.4	1.3–1.6	<0.001	1.3	1.1–1.5	1.2×10^−4^
**Occupation (Compared with "others")**						
**Farmer**	3	2.7–3.3	<0.001	1.1	1–1.3	0.18
**Age (Compared with "0–17")**						
**18–34**	1	0.6–1.5	0.9	1.1	0.6–1.7	0.9
**34–59**	1.3	0.9–2	0.18	1.4	0.9–2.3	0.17
**>60**	3.3	2.2–5	<0.001	3.3	2–5.5	<0.001
**Treatment history (Compared with "new cases")**						
**Previously treated cases**	7.6	6.8–8.6	<0.001	6.6	5.8–7.4	<0.001
**Region (Compared with "Southeast Districts")**						
**Urban Districts**	0.3	0.3–0.4	<0.001	0.32	0.25–0.42	<0.001
**New Urban Development Districts**	2	1.5–2.5	<0.001	1.3	1–1.8	0.04
**Northeast Districts**	2.7	2.1–3.5	<0.001	1.8	1.4–2.4	<0.001

In multiple variable analysis, MDR/RR-TB notification was not significantly associated with occupation. Other risk factors associated with MDR/RR-TB in single variable analysis were still correlated in multiple variable analysis.

## Discussion

The MDR/RR-TB notification rate has increased significantly since 2010 in Chongqing. There were several reasons for this increase of MDR/RR-TB notification rate. One was that the foundation of MDR/RR-TB control system remained weak before 2009. The detection and treatment of MDR/RR-TB was not included in TB control plan of Chongqing in that period, and the MDR/RR-TB cases were not recorded in the TB surveillance system before 2010. This has contributed to the low MDR/RR-TB notification rate in early stage. Change began in 2009 by a project supported by the Bill and Melinda Gates Foundation [4]. This project aimed to improve MDR/RR-TB diagnosis and treatment in some counties and districts in 2011, including the following measures: the introduction of rapid molecular diagnosis, promotion of investment by local medical insurance and the foundation covering 90% of the medical cost, standard MDR/RR-TB treatment regimen and management, improved MDR/RR-TB detection, and establishment of cooperative mechanism between hospitals and CDC. Driven by this project, government funding for MDR/RR-TB has been rising year by year, and a sustainable financing mechanism has been established. After this project, the reimbursement rate of medical insurance for MDR/RR-TB has been increased to 90% with inpatient and outpatient care. The MDR/RR-TB screening has been financed by government since 2012, and rapid molecular diagnosis equipment was distributed to some laboratories. The scope of MDR/RR-TB screening has been expanded to all notified bacteriological positive TB cases. The laboratory diagnostic ability has been improved with continual training and supervision. The MDR/RR-TB reporting has been required in the electronic national TB surveillance system since 2010. With these improved measures, more and more MDR/RR-TB cases were notified. In 2017, the MDR/RR-TB notification rate was 2.1 cases per 100,000 population. But there still remained a detection gap between the number of notified MDR/RR-TB cases and the estimate number of MDR/RR-TB cases in new TB cases. Screening should be further strengthened with more investment by government.

In Chongqing, the MDR/RR-TB notification rate of male was significantly higher than the rate of female, and male was more likely to develop MDR/RR-TB than female according to the risk factor analysis. But gender impact on MDR/RR-TB was reported differently in different regions. There was no association with gender in several studies [8–13]. In some regions of China, studies showed that female was more likely to develop MDR/RR-TB [14, 15]. Socioeconomic condition could be one of the reasons for this difference. Chongqing was a city with a large poor rural population and many underdeveloped regions. Many men needed to go out to developed regions to work and support their families. Inadequate treatment and irregular management were more likely to happen in these cases with TB, which were important risk factors leading to MDR/RR-TB [16–18].

Our risk factor analysis based on MDR/RR-TB screening data showed that only over-60-year age group was the risk factor associated with MDR/RR-TB, which meant over-60-year age group were more likely to develop MDR/RR-TB. But the MDR/RR-TB notification rate of over-60-year age group (OR, 1.5; 95% CI, 1.2–1.8) was significantly lower than the rate of 18–34 age group (OR, 2.1; 95% CI, 1.9–2.4) from 2010 to 2017. This contradiction may indicate that the MDR/RR-TB notification in over-60-year age group was under-reported. Other study also showed that aging was a risk factor of MDR/RR-TB [11]. The correlation between aging and MDR/RR-TB might be due to socioeconomic status and common diseases of the elderly, like diabetes [19, 20]. The screening for MDR/RR-TB in elder TB cases should be strengthened.

The MDR/RR-TB notification rate of farmers was higher than the rate of all other occupations and increased to 4.1 cases per 100,000 population in 2017. Our study showed that the occupation of farmer was associated with MDR/RR-TB, and some studies in other regions of China also showed that farmer was a risk factor [21]. In Chongqing, farmer was a poor occupation. Although the income of farmer has gone up rapidly in recent years, the socioeconomic status of them was still low. According to Chongqing Statistical Yearbook, the annual income of farmers was less than half of urban residents. MDR/RR-TB was closely associated with poverty [22–24]. Medical services for MDR/RR-TB were often poorly accessible in rural areas [25].

The MDR/RR-TB notification rate of Urban Districts has been significantly higher than other three regions. But the region of Urban Districts was a protective factor in the risk factor analysis for regional disparity. This was another contradiction in our study which may indicate that the detection of MDR/RR-TB was underestimated in other three regions. According to Chongqing Statistical Yearbook, the GDP per capita of Urban Districts was about two times higher than the level of other three regions from 2010 to 2017. The two largest prefectural designated hospitals, the provincial reference laboratory and provincial TB prevention and control institution were all located in Urban Districts. The lower socioeconomic status and weakness in health resources may lead to the under notification of MDR/RR-TB in other three regions.

Great progress has been made in the treatment of MDR/RR-TB. The treatment success rate of MDR/RR-TB increased significantly from 20% (95% CI, 2.9%-43%) in 2011 cohort to 61% (95% CI, 53%-70%) in 2015 cohort. The treatment success rate was 41% in 2014 cohort for whole China [2]. Cooperation between hospitals and CDC has proved successful. But 21.4% of notified cases were not involved in treatment, and 20.1% of them had not treatment outcome information reported. Even if medical insurance has covered 90% of the medical cost of MDR/RR-TB treatment, some poor cases still could not afford the out-of-pocket expenses, and were not involved in treatment because of poverty [26]. The information loss of treatment outcome indicated that cooperation between hospitals and CDC needed to be strengthened in the fields of information delivery and patient management.

From 2010 to 2017, the drug resistance rate for RFP in new TB cases was 9% (95% CI, 8.1%-10%), and this rate in previously treated TB cases was 42% (95% CI, 39.4%-44%) in Chongqing. This rate was only 4.1% in new TB cases and 19% in previously treated TB cases globally [2]. Chongqing is facing high prevalence of MDR/RR-TB, especially in previously treated TB cases. The drug resistance rate for RFP increased significantly from 7.1% (95% CI, 1.9%-12.2%) in 2010 to 25.9% (95% CI, 23.3%-28.5%) in 2017. There were two possible reasons. One was that screening in high-risk groups has been effectively implemented in recent years, and more and more MDR/RR-TB cases were notified. A significant improvement in laboratory diagnostic capacity may be the other one.

This study has limitations. The epidemiological survey for MDR/RR-TB has not been implemented in recent years, so the routine surveillance data have been analyzed in our study. The estimated number of MDR/RR-TB cases has been evaluated by the most recent MDR/RR-TB proportions in new and previously treated TB cases, which were measured based on national survey in 2013, and the provincial data were not available because the surveillance survey of drug-resistant tuberculosis has never been conducted in Chongqing. The age groups could only be divided into four age groups according to Chongqing Statistical Yearbook. The treatment success rate of 2010 cohort could not been shown because all patients did not receive the treatment except one who had not treatment outcome information reported in 2010 (Fig 7). The MDR/RR-TB cases who died from causes other than TB have not been recorded in the national electronic TB surveillance system until now, so we could not take into account these cases in calculation of the treatment success rate. In the missing data analysis of the treatment outcome, neither of the mean ages of two groups is within the other confidence interval, but the Z-statistic was 1.7 and the p-value was 0.09.

In conclusion, MDR/RR-TB control over the years has been effective in Chongqing, and more and more MDR/RR-TB cases have been notified and treated. But the prevalence of MDR/RR-TB is still high, and facing the challenges including detection and treatment gaps, the regional disparity due to socioeconomic status, the under-notification in elderly people, and the high notification rate in farmers. Sustained government financing and policy support should be guaranteed to ensure universal access to effective MDR/RR-TB medical care.
